# Higher levels of markers for early atherosclerosis in anti-citrullinated protein antibodies positive individuals at risk for RA, a cross sectional study

**DOI:** 10.1007/s00296-024-05659-5

**Published:** 2024-07-16

**Authors:** Helma J. Hinkema, Johanna Westra, Suzanne Arends, Elisabeth Brouwer, Douwe J. Mulder

**Affiliations:** 1 Department of Rheumatology and Clinical Immunology, University Medical Center Groningen, P.O. Box 30001, Groningen, 9700 RB The Netherlands; 2 Department of Internal Medicine, University Medical Center Groningen, Groningen, The Netherlands

**Keywords:** Anti citrullinated protein antibodies, Biomarkers, Cardiovascular disease, Rheumatoid arthritis

## Abstract

**Objective:**

To identify differences in levels of serum biomarkers associated with atherosclerosis between anti-citrullinated protein antibodies (ACPA) positive groups.

**Methods:**

Cross-sectional data were used from the Dutch Lifelines Cohort Study combined with data derived from RA risk and early RA studies conducted at the University Medical Center Groningen (UMCG).

Serum biomarkers of inflammation, endothelial cell activation, tissue remodeling and adipokine, which were previously associated with atherosclerosis, were measured with Luminex in four ACPA positive groups with different characteristics: without joint complaints, with joint complaints, RA risk and early RA groups.

**Results:**

Levels of C-reactive protein (CRP), Interleukin-6 (IL-6), Tumor Necrosis Factor Receptor 1 (TNFR1) and vascular endothelial growth factor (VEGF) were significantly higher in the RA risk and early RA groups compared to the joint complaints and the no joint complaints groups. The difference remained statistically significant after correcting for renal function, smoking and hypertension in multivariate logistic regression analysis, with focus on ACPA positive with joint complaints group versus RA risk group: CRP OR = 2.67, *p *= 0.033; IL-6 OR = 3.73, *p* = 0.019; TNFR1 OR = 1.003, *p* < 0.001; VGEF OR = 8.59, *p* = 0.019.

**Conclusion:**

Individuals at risk for RA have higher levels of inflammatory markers and VEGF, which suggests that they might also have a risk of higher cardiovascular disease (CVD); however, this does not apply to individuals with ACPA positivity with self-reported joint complaints or without joint complaints only. Therefore, it is important that individuals with RA risk are referred to a rheumatologist to rule in or out arthritis/development of RA and discuss CVD risk.

## Introduction

It is widely accepted nowadays that rheumatoid arthritis (RA) is associated with a higher incidence of cardiovascular disease [1–4]. Several studies showed that cardiovascular events can already occur at a higher than expected rate shortly after the first symptoms of RA [5], possibly related to inflammation. Chronic inflammation is considered to play an important role in atherosclerosis, contributing to a higher risk of cardiovascular disease [6–9]. Endothelial cell activation and dysfunction are considered to be the first steps in this inflammatory process. Several serological biomarkers are described to have an association with subclinical atherosclerosis and inflammation [7, 10].

There are several important serological proteins that may play a role in the development of atherosclerosis. Monocyte chemoattractant protein-1 (MCP-1), matrix metalloproteinases (MMPs) and vascular endothelial growth factor (VEGF) are thought to promote plaque development [11–14]. Atherosclerosis is characterized by atherosclerotic plaques, which are in general composed of a lipid core covered with a fibrous cap, mainly consisting of extracellular matrix, collagen fibers, smooth muscle cells and macrophages [15].

Also inflammatory markers like interleukin 6 (IL-6) and high sensitive c-reactive protein (CRP), together with interferon gamma-induced protein 10 (IP-10), tumor necrosis factor receptor 1 (TNFR1) and chitinase-3-like protein 1 (CHI3L1), also known as YKL-40 are described to play a role in atherosclerotic plaque formation [16–24].

Next to inflammatory markers and endothelial cell activation markers also adipokines, such as leptin, appear to play a role in cardiovascular disease [25]. Leptin levels are positively correlated with body mass index (BMI) and obesity [25].

Finally, in previous studies, a relation was suggested between presence of anticitrullinated protein antibodies (ACPA) and subclinical atherosclerosis estimated by intima media thickness in RA patients [26, 27]. In line with these findings, Lopez –Longo et al. found in their RA cohort that ACPA were associated with the development of ischemic heart disease, irrespective of ACPA titers and independent of traditional cardiovascular risk factors [28].

The aim of this study was to identify differences in levels of serum biomarkers associated with atherosclerosis between ACPA positive groups with different characteristics: without joint complaints, with joint complaints, RA risk and early RA groups.

In this comparison, we primarily focused on differences between ACPA positive individuals with joint complaints versus RA risk patients. The difference between these two groups is that the joint complaints (based on questionnaires) group is derived from the population based lifelines cohort and the RA risk group is based on individuals who were diagnosed by a trained rheumatologist as having seropositive arthralgia at risk for developing rheumatoid arthritis [29]. Presence of musculoskeletal symptoms is very prevalent in individuals visiting their general practitioner, however only a small number of them are suspected of having arthritis [30]. We hypothesize that patients in the RA risk group have raised biomarkers associated with atherosclerosis and thereby possibly already have an increased risk for cardiovascular disease in comparison with ACPA positive individuals with only joint complaints.

## Materials and methods

For this cross-sectional study, blood samples were used from the Dutch Lifelines Cohort Study combined with blood samples from previous clinical studies conducted in the University Medical Center Groningen (UMCG) for measurement of serological biomarkers associated with atherosclerosis.

## Participants

We included four ACPA positive groups with different characteristics, comprising an ACPA positive without joint complaints group (*n* = 95), an ACPA positive with joint complaints group (*n* = 83), both from Lifelines, an ACPA positive RA risk group (*n* = 28) and an ACPA positive early RA group (*n* = 33), to enable identifying differences in serum biomarkers associated with atherosclerosis. As all groups are ACPA positive, we will from now on refer to the groups as no joint complaints group for the ACPA positive without joint complaints group, joint complaints group for the ACPA positive with joint complaints group, RA risk group for the ACPA positive RA risk group and early RA for ACPA positive early RA group.

### No joint complaints group and joint complaints group

Lifelines data were used from participants who had ACPA values measured at baseline irrespective of specific symptoms as previously described, thereby enabling us to compose a ACPA positive group without joint complaints [31]. Lifelines is a multi-disciplinary prospective population-based cohort study examining in an unique three-generation design health and health-related behaviors of 167.729 persons living in the North of the Netherlands. It employs a broad range of investigative procedures in assessing the biomedical, socio-demographic, behavioral, physical and psychological factors which contribute to the health and disease of the general population, with a special focus on multi-morbidity and complex genetics [32]. Information on application and data access procedure is summarized on https://www.lifelines.nl*.* The Lifelines protocol was approved by the UMCG Medical ethical committee in 2007 under number 2007/152.

To define ACPA positive participants with or without joint complaints, data from the connective tissue disease (CTD) screening questionnaire (CSQ) had to be available. The joint complaints group was composed using the CSQ, using eight questions comprising joint stiffness in the morning, joint complaints for > 3 months, duration of morning stiffness lasting at least 1 h for > 6 weeks, presence of joint complaints in the same joints on both sides of the body, joint pain, pain in the hand or wrist, swollen hands or wrists and difficulty with making a fist. The baseline measurement took place in 2012–2013 and the follow-up assessment took place approximately 2 years after baseline assessment [33]. When participants reported no joint complaints in the CSQ they were assigned to the no joint complaints group. When they reported joint complaints in the CSQ, by answering positive on 1 or more of 8 above mentioned CSQ questions, they were assigned to the joint complaints group.

Individuals with RA were excluded as defined by the following: individuals with self-reported RA and individuals with medication use for indication of rheumatism and visiting a medical specialist within the last year [31]. In the joint complaints group and no joint complaints group people were allowed to use over the counter painkillers like acetaminophen and/or non-steroidal anti-inflammatory drugs (NSAIDs).

### RA risk group

All the patients attended the outpatient clinic of the University Medical Center Groningen after they were referred to the early arthritis clinic or early arthritis recognition clinic by their general practitioner. They were selected based on presence of arthralgia (tender joints count (TJC) ≥ 1, but no diagnosis of arthritis (swollen joint count (SJC) = 0) in combination with positive rheumatoid factor and/or ACPA antibodies. The diagnosis of seropositive arthralgia was made by a trained rheumatologist. Patients had their first assessment in 2012–2015 as described previously [34]. For this study, only the ACPA positive individuals were selected with stored serum samples. The study was approved in 2011 by the local ethical board in the UMCG and all patients provided written informed consent (Metc 2011.306). The patients did not receive disease modifying anti rheumatic drugs (DMARDs) and were treated with non-steroidal anti-inflammatory drugs (NSAIDs) only.

### Early RA group

The early RA group consisted of patients fulfilling the 1987 or 2010 American College of Rheumatology (ACR) criteria for RA. The patients were included at the time of diagnosis. The baseline assessments took place on the same day as patients were diagnosed with RA and were informed on the DMARD effects and side effects and received a prescription at our outpatient clinic. Therefore the samples were taken before start of the DMARD treatment, however they were allowed to use NSAIDs before the diagnosis was made.

All the patients attended the outpatient clinic of the UMCG. The study was approved in 2009 by the local ethical board of the UMCG and all patients provided written informed consent (Metc 2009–118). Patients were recruited between 2010 and 2012 [34]. For this study, only ACPA positive patients were selected with stored serum samples.

### Clinical assessments

Regarding the Lifelines data, characteristics were acquired at baseline visit when serum blood samples were stored, including measurements of BMI, blood pressure, and extensive questionnaire comprising information regarding smoking behavior (current smoking), presence of cardiovascular disease and family history for cardiovascular disease. Hypertension was defined as a systolic blood pressure > 140 mmHg and/ or diastolic blood pressure > 90 mmHg and/ or presence of hypertension derived from the questionnaires. Cardiovascular disease was defined as self-reported presence of cardiovascular disease comprising non-fatal major cardiovascular events, including myocardial infarction, percutaneous transluminal coronary angioplasty surgery, coronary artery bypass grafting surgery and ischemic or haemorrhagic stroke.

Regarding the RA risk patients and early RA patients, hypertension was defined as a systolic blood pressure > 140 mmHg and/ or diastolic blood pressure > 90 mmHg. Diabetes mellitus, was defined as presence of diabetes mellitus as diagnosis in the medical recordings and/or use of antidiabetic drugs.

### Sample analysis for anticitrullinated protein antibodies

Serum samples were stored at − 20 °C until analysis. ACPA detection was performed by measuring IgG anti- CCP2 on the Phadia-250 analyser, with a measuring range (detection limit, upper limit) for ELiA CCP from 0.4 EliA U/ml to ≥ 340 EliA U/ml. ACPA levels ≥ 6,2 U/ml were considered positive, as described previously [31].

### Sample analysis for serological biomarkers associated with atherosclerosis

All samples for the 4 different groups were stored at – 20 ⁰C until analysis. We performed a cross-sectional analysis based on samples from 4 different groups;

1. Lifelines cohort: ACPA positive without joint complaints participants (*n* = 95), samples at first visit taken 2012–2013.
2. Lifelines cohort: ACPA positive with joint complaints participants (*n* = 83), samples at first visit taken 2012–2013.
3. Outpatient clinic: ACPA positive RA risk patients (*n* = 28), samples taken at inclusion 2012–2015.
4. Outpatient clinic: ACPA positive early RA group (*n* = 33), samples taken at inclusion 2010–2012 before DMARD treatment (which was started the same or next day after the sample was taken).

(see Fig. 1).

**Fig. 1:**
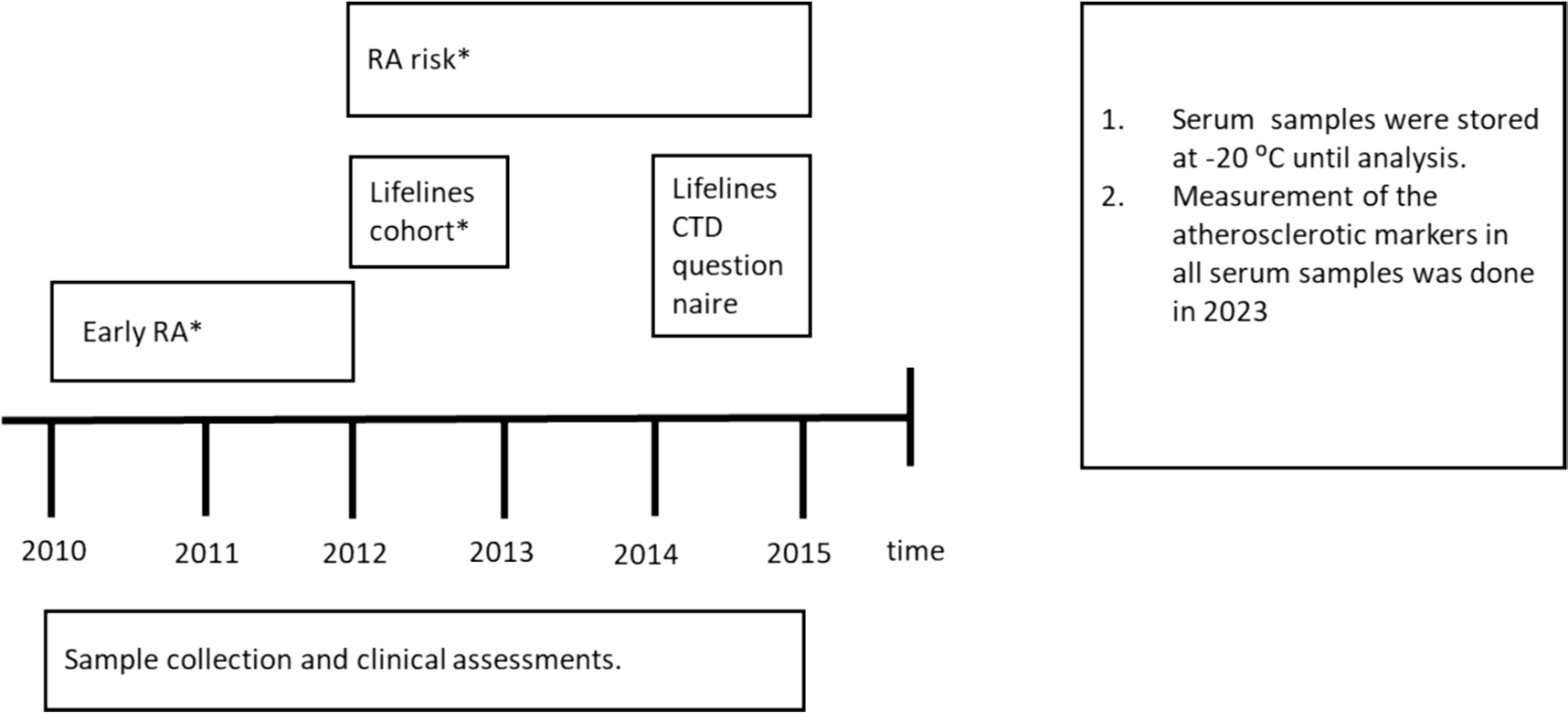
Overview of timeline of inclusion of participants and serum samples for measurement atherosclerosis markers. *CTD* connective tissue disease. * Inclusion of participants with first assessments and sample collection

The markers of inflammation (IL-6, TNF-R1, IP-10), endothelial cell activation (VEGF), tissue remodeling (YKL-40, MMP-3, MCP-1) and adipokine (leptin) were determined with Human premix Magnetic Luminex screening assay kits (R&D Systems, Abingdon, UK) according to the manufacturer’s instructions. Serum dilution was 1:2 [34].

High sensitivity CRP (hsCRP) was performed with Duo set ELISA (R&D Systems) according to the manufacturer’s instructions.

### Statistical analysis

Statistical analysis was performed with IBM SPSS Statistics 25. Results were expressed as number of subjects (percentage), mean ± SD or median (IQR) for categorical, normally and non-normally distributed data, respectively. One way ANOVA or Kruskal Wallis test followed by Mann Whitney *U* test was used as appropriate to compare characteristics and serum levels biomarkers between participants of the 4 groups: 1. no joint complaints group, 2. joint complaints group, 3. RA risk group and 4. early RA group. Subsequently, binary logistic regression analysis was performed to compare serum levels of biomarkers in the RA risk group versus the joint complaints group. Multivariable binary logistic regression analysis was performed to correct for renal function, smoking and hypertension. Because of skewed distribution of residuals of IL-6, CRP and VEGF, we performed a sensitivity analysis with log-transformation. Correlation between serum levels of biomarkers was performed using Pearson’s or Spearman’s correlation coefficient for normally and non-normally distributed data, respectively.

## Results

In total, 40,136 lifelines participants had baseline ACPA levels available, from which 401 (1,0%) had an ACPA level ≥ 6.2 U/ml. 311 participants were ACPA positive without (possible) RA and 178 (57.8%) of these individuals had responded to the CSQ questionnaire [33].

Of the 178 remaining ACPA positive individuals, 83 reported joint complaints, resulting in 95 individuals in the no joint complaints group and 83 individuals in joint complaints group. Furthermore, we included 28 individuals in the RA risk group and 33 individuals in the early RA group. The baseline characteristics of the groups are summarized in Table 1.

**Table 1:** Baseline characteristics of ACPA positive individuals

	no joint complaints (*n* = 95)	joint complaints (*n* = 83)	RA risk (*n* = 28)	Early RA (*n* = 33)	*p*-value
Age (years), mean (± SD)	43 ± 12	49 ± 10	47 ± 15	58 ± 14	< 0.001
Gender (female), *n* (%)	52 (55)	59 (72)	21 (75)	26 (79)	0.005
BMI (kg/m2) median (IQR)	24.4 (22–27)	25.2 (23–28)	24 (23–29)	26 (23–30)	NS
Obesity, *n* (%)	< 10 (≤ 10)*	< 10 (≤ 10)*	5(18)	9 (27)	0.008
Systolic blood pressure (mmHg), median (IQR)	125 (117–134)	122 (114–133)	120 (120–130)	140 (130–160)	< 0.001
Diastolic blood pressure (mmHg) median (IQR)	72 (76–80)	74 (66–81)	80 (70–80)	84 (80–90)	< 0.001
Hypertension, (yes), *n* (%)	23 (24)	34 (41)	5 (18)	15 (46)	0.012
Creatinine (umol/l), median (IQR)	71 (66–81)	70 (63–79)	66 (56–70)	62 (55–73)	< 0.001
Smoking, (yes) n, (%)	19 (20)	16 (19)	17 (60)	4 (12)	0.015
T2DM, (yes) *n* (%)	0 (0)	< 10 (< 10)	1 (4)	0 (0)	NS
ACPA (IU/ml)	17 (8–61)	19 (9–66)	111 (46–330)	304 (98–340)	< 0.001

Baseline Characteristics of included participants. *BMI* Body Mass Index, *T2DM* Type 2 Diabetes Mellitus, *CVD* cardiovascular disease, *SD* Standard Deviation, *IQR* interquartile range

^*^for privacy reasons, to avoid possible traceability of participants, it is not allowed according to the Lifelines protocol to publish the exact number of individuals in the no joint complaints group and joint complaints group

Overall, there were significant differences between the 4 groups in age, gender, obesity, blood pressure, hypertension, renal function, smoking and ACPA levels.

There were fewer females (55%) in the no joint complaints group compared to the other three groups (72–77%). When comparing the no joint complaints group with the joint complaints group, the individuals in the joint complaints group were significantly older and they more often had hypertension. The RA risk group had more smokers compared to the joint complaints group and the early RA group.

Although blood pressure was not significantly different, the joint complaint group had more individuals with hypertension and showed a higher creatinine level. Early RA patients were older, more often obese and had higher blood pressure compared to the other 3 groups. Also, early RA patients more often had hypertension compared to the RA risk group. ACPA levels were significantly higher in the RA risk group and early RA group.

**Fig. 2:**
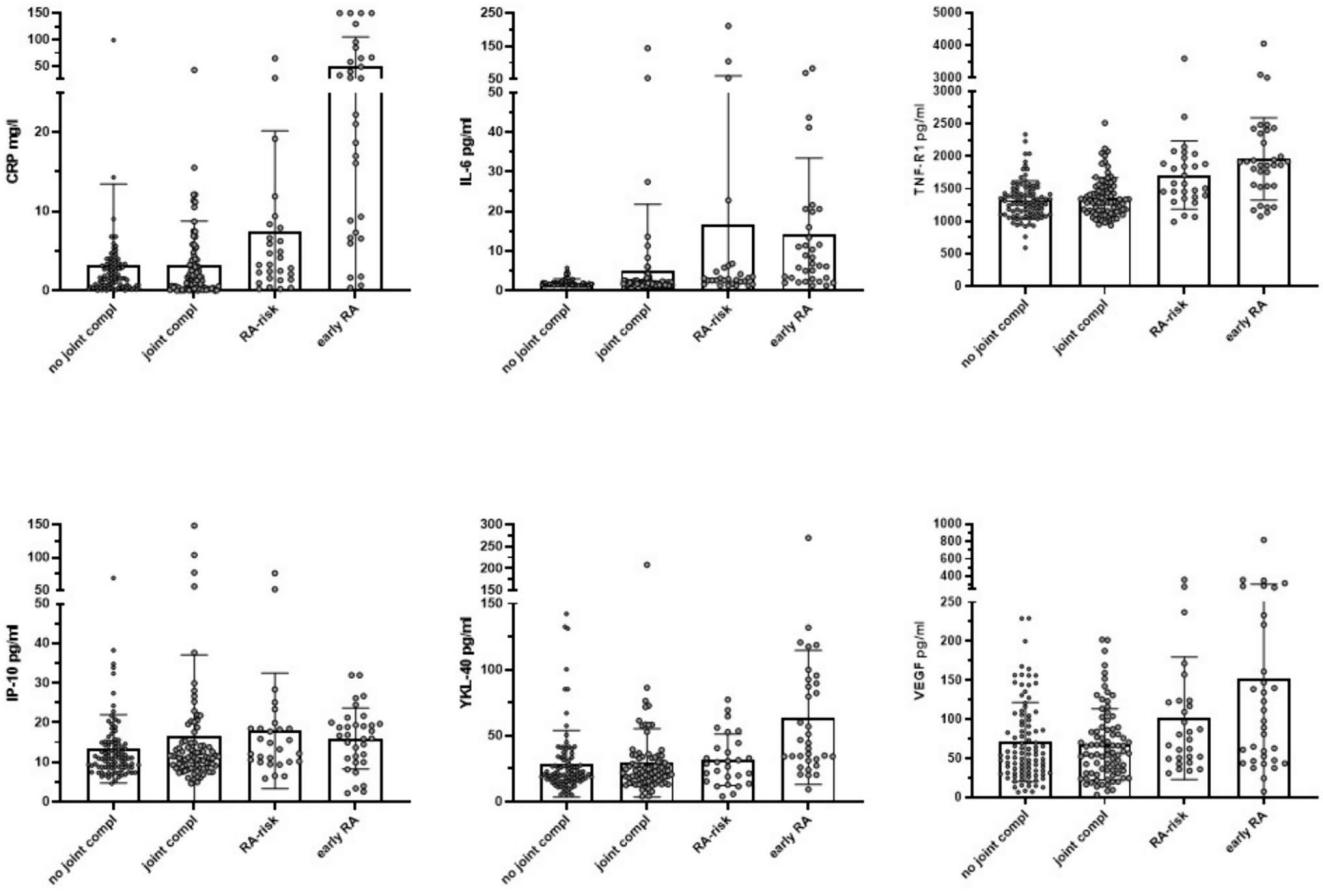
Differences in serum biomarkers CRP, IL-6, TNF-α, IP-10, YKL-40, and VEGF between the no joint complaints group, joint complaints, RA risk group for the ACPA positive RA risk group and early RA group

CRP, IL-6, TNF-R1, IP-10, VEGF levels were significantly higher in the RA risk and early RA group compared to the no joint complaints group and joint complaints group (Fig. 1). YKL-40 levels were significantly higher in the early RA group compared to no joint complaints group, joint complaints group and RA risk group (Fig. 1). The leptin levels were higher in the joint complaint group compared to the no joint complaint group. When comparing the RA risk group with the early RA group, the early RA group had significantly higher CRP (*p* < 0.001), IL-6 (*p* = 0.016) and YKL-40 (*p* < 0.001) levels. (Fig. 2).

In the RA risk group, a positive correlation was observed between VEGF and CRP levels (rho = 0.533, *p* = 0.003) and between VEGF and IL-6 levels (rho = 0.467, *p* = 0.012).

In the early RA group, there was a positive correlation between VEGF and anti TNFR1 levels (rho = 0.412, *p* = 0.017) and between VEGF and CRP levels (rho = 0.560, *p* = 0.001). There was no significant correlation between the ACPA levels and levels of CRP, IL-6, anti TNFR-1, IP-10, VEGF, and leptin.

As mentioned, we were specifically interested in differences between RA risk patients and joint complaints groups. As shown in Table 2, CRP, IL-6, TNF-R1, VEGF levels were significantly higher in RA risk group compared to joint complaint group. These results were confirmed in univariable logistic regression analysis, which remained significant after correcting for renal function, smoking and hypertension in multivariable logistic regression analysis (Table 3). Log-transformed IL-6 and CRP levels also remained significantly higher after correcting for renal function, smoking and hypertension (Table 3).

**Table 2:** Serum levels of biomarkers between the joint complaints group and RA risk group

	joint complaints (*n* = 83)	RA risk (*n* = 28)	*p*-value
Markers of inflammation			
CRP (mg/l) median (IQR)	1.7 (0.5–3.8)	3.3 (1.4–7.6)	0.008
IL-6 (pg/ml) median (IQR)	1.9 (1.4–2.35)	2.8 (1.8–5.5)	0.002
TNF-R1(pg/ml) median (IQR)	1312 (1146–1528)	1579 (1379–1953)	< 0.001
IP-10 (pg/ml) median (IQR)	11.4 (8.5–14.9)	14.1 (10.1–18.5)	NS
Markers of endothelial cell activation			
VEGF (pg/ml) median (IQR)	57 (31–87)	77 (49–123)	0.034
Tissue remodeling markers			
Chit 3-like1 / YKL-40 (ng/ml) median (IQR)	23.6 (16.8–33.6)	29.4 (16.3–43.5)	NS
MMP-3 (ng/ml) median (IQR)	9.1 (6.6–12.1)	9.1 (6.4–11.7)	NS
MCP-1 (pg/ml) median (IQR)	319 (257–398)	280 (222–480)	NS
Adipokine			
Leptin (ng/ml) median (IQR)	9.8 (3.7–15.5)	7.9 (3.5–15.6)	NS

Serum levels of biomarkers between the joint complaints group and RA risk group

*RA* rheumatoid arthritis, *IQR* interquartile range

**Table 3:** Univariable and multivariable logistic regression analyses corrected for renal function, smoking (yes/no) and hypertension (yes/no)

RA risk group (*n* = 28) OR (95% CI)				
	Univariable*	*p*-value	Multivariable*	*p*-value
Markers of inflammation				
CRP	2.809 (1.277–6.181)	0.010	2.673(1.081–6.613)	0.033
IL-6	3.658 (1.357–9.856)	0.010	3.732 (1.247–11.164)	0.019
TNF-R1 Markers of endothelial cell activation	1.002 (1.001–1.004)	< 0.001	1.003 (1.001–1.004)	< 0.001
VEGF	7.274 (1.547–34.195)	0.012	8.591 (1.415–52.146)	0.019

Reference group: ACPA positive with joint complaints group (*n* = 83)

^*^using the log transformed for CRP, IL-6 and VEGF because of skewed distribution of residuals

## Discussion

In this study including four groups of ACPA positive individuals with different characteristics, we found higher levels of inflammatory markers (CRP, IL-6 and TNFR1) and endothelial cell activation marker (VEGF) in the RA risk patients compared to individuals with or without joint complaints. This indicates the presence of subclinical inflammation, which is in line with other studies showing subclinical inflammation in the pre-clinical phase of RA [34, 35]. As expected, the levels of inflammation were even higher in the early RA group, reflected by higher levels of CRP, IL-6 and TNFR1. Nidorf et al. suggested that subclinical inflammation, reflected by slightly higher levels CRP appears to be of relevance in cardiovascular disease risk [8, 36]. They showed in the LoDoCo trial that targeting an inflammatory pathway with colchicine, next to standard secondary prevention, appeared effective for the prevention of cardiovascular events in patients with stable coronary disease [8]. This supports the idea that subclinical inflammation in RA risk patients might be indicative for a higher cardiovascular disease risk. Furthermore, CRP and IL-6 are described to be elevated in atherosclerosis and therefore the higher levels of CRP and IL-6 in the RA risk group might partly be explained by the underlying atherosclerotic process.

The higher VEGF levels in the early RA group compared to the no joint complaints and joint complaints groups is in line with previous studies [13, 37]. The VEGF levels were also higher in the RA risk group compared to the no joint complaint group and joint complaint group. VEGF levels are described to be upregulated by pro-inflammatory cytokines [37]. Endothelial cell activation induces expression of pro-inflammatory cytokines [38]. In our study, we found a positive correlation between VEGF and anti-TNFR1 levels and between VEGF and CRP levels in the early RA group. Also a positive correlation between VEGF and CRP levels and between VEGF and IL -6 levels in the RA risk group were seen, which is supportive for the described relation between VEGF and pro-inflammatory cytokines. VEGF is an important pro-angiogenic mediator [13] and is suggested to play a role in the development of vulnerable atherosclerotic plaques [14], however, also protective effects by promoting collateral vessel formation are described [39]. As VEGF, CRP, IL-6 and TNF-R1 are regarded as biomarkers for atherosclerosis this implies that patients in the RA risk group might already have a higher cardiovascular disease risk.

YKL-40 levels were not significantly different between the joint complaints and RA risk group, however, we did find higher levels of YKL-40 in the early RA group. Therefore, YKL-40 might be a marker which is present later on in the process. This is in accordance with the finding that YKL-40 is described to be related to disease activity and joint destruction in RA [40, 41].

Although leptin levels have been described to play a role in coronary heart disease and correlate with obesity [25, 42, 43], we did not find signficant different levels of leptin between the joint complaint group and the RA risk group. This might be due to the absence of difference between presence of manifest cardiovascular disease and obesity in these groups. Furthermore, the leptin levels were significinantly lower in the early RA group compared to the joint complaint group and RA risk group. Conflicting results have been described concerning the association between leptin levels and cardiovascular disease. Multiple studies have described higher leptin levels as a risk factor for cardiovascular disease in the general population and in RA patients with a positive correlation with BMI [25, 42, 43]. However, Curtis et al. found lower levels of leptin and a lower BMI in RA patients with a CVD event compared to RA patients without a cardiovascular event and suggested that inflammation in RA may contribute to weight loss and mortality [44].

Van de Stadt et al. showed that titers of ACPA levels increased approximately 2–4 years before diagnosis of RA [45] and ACPA levels have been described to have a more pro-inflammatory profile in RA risk [46]. However, in the absence of a ACPA negative control group, we cannot conclude whether ACPA positivity alone can account as a possible read out for the presence of subclinical inflammation and endothelial disfunction. When taking into account the lack of difference in the no joint complaints group and the joint complaints group as well as the presence of higher inflammation levels in the RA risk group and early RA group, ACPA positivity alone is not enough as a read out and more steps towards developing RA are needed, which is in line with our previous study [47]. Furthermore, this is supported by the absence of correlation between ACPA levels and levels of inflammatory markers, however this might be influenced by the relatively small groups.

In contrast with Rantapaa-Dahlqvist et al., we did not find higher levels of MCP-1 in the RA risk and early RA group. These differences might be due to relatively low numbers in both studies and difference in sample analysis (Luminex vs Elisa) [48].

The main limitation of this study is the cross sectional study design with relatively small groups. Another limitation is that there was no well described definition of cardiovascular disease in the RA risk and early RA group. Furthermore, we did not have data regarding intima media thickness, which is considered an indicator of generalized subclinical atherosclerosis [49, 50]. Therefore we were not able to correlate the atherosclerotic biomarkers with intima media thickness a sign of increased atherosclerosis. Furthermore the atherosclerosis markers were measured in 2023 in serum samples which were all stored in the same way in – 20 ⁰C freezers. The presence of joint complaints in Lifelines is based on questionnaires filled in by participants. This in contrast to the RA risk group and early RA group, in which the joints were examined by a rheumatologist. Since joint complaints reported by patients can be caused by trauma, degenerative diseases and pain syndromes, the clinical expertise of the rheumatologist is important to rule in or rule out arthritis and to identify individuals with RA risk, however, not all individuals which are identified as RA risk will develop RA in the future.

In conclusion, we found that ACPA positive RA risk patients already have higher inflammatory markers and higher VEGF levels in comparison to ACPA positive individuals with or without joint complaints. This indicates that individuals with RA risk might already have a higher cardiovascular risk. These findings suggest that clinical expertise toward the development of RA is needed and that presence of joint complaints reported in questionnaires is not associated with an increased cardiovascular risk in ACPA positive individuals. However, further research is needed to establish whether presence of subclinical inflammation and presence of endothelial cell activation markers are related to a higher cardiovascular disease risk in RA risk patients. Therefore, prospective studies collecting data regarding early biomarkers in relation to measures of subclinical atherosclerosis markers and cardiovascular events in RA risk patients could be supportive. Meanwhile, it is important that individuals with RA risk are referred to a rheumatologist to determine arthritis/development of RA and discuss CVD risk. Early detection could provide an opportunity for prevention of clinical manifest cardiovascular disease. In line with these findings cardiovascular risk management is important in patients with newly diagnosed with rheumatoid arthritis as in longstanding RA patients.

## Data availability statement

Data are available from the University of Groningen-UMCG Institutional Data Access for researchers who meet the criteria for access to confidential data. The local ethics committees of the University Medical Center Groningen (UMCG) will maintain the ethical restrictions of the data. The Data Protection Officer of the UMCG will maintain the legal restrictions and appropriate codes of conduct. Permission is required prior to access. Data requests can be sent to Research Data Office University of Groningen: researchdata@rug.nl. Concerning lifelines data: Data may be obtained from a third party and are not publicly available. Researchers can apply to use the Lifelines data used in this study. More information about how to request Lifelines data and the conditions of use can be found on their website: https://www.lifelines.nl/researcher/how-to-apply.

## Data Availability

Data are available from the University of Groningen-UMCG Institutional Data Access for researchers who meet the criteria for access to confidential data. The local ethics committees of the University Medical Center Groningen (UMCG) will maintain the ethical restrictions of the data. The Data Protection Officer of the UMCG will maintain the legal restrictions and appropriate codes of conduct. Permission is required prior to access. Data requests can be sent to Research Data Office University of Groningen: researchdata@rug.nl. Concerning lifelines data : Data may be obtained from a third party and are not publicly available. Researchers can apply to use the Lifelines data used in this study. More information about how to request Lifelines data and the conditions of use can be found on their website: https://www.lifelines.nl/researcher/how-to-apply.

## Declarations

### Open Data Sharing

N/A.
